# Unraveling the Complexities of Oxidative Stress and Inflammation Biomarkers in Obstructive Sleep Apnea Syndrome: A Comprehensive Review

**DOI:** 10.3390/life14040425

**Published:** 2024-03-22

**Authors:** Salvatore Lavalle, Edoardo Masiello, Giannicola Iannella, Giuseppe Magliulo, Annalisa Pace, Jerome Rene Lechien, Christian Calvo-Henriquez, Salvatore Cocuzza, Federica Maria Parisi, Valentin Favier, Ahmed Yassin Bahgat, Giovanni Cammaroto, Luigi La Via, Caterina Gagliano, Alberto Caranti, Claudio Vicini, Antonino Maniaci

**Affiliations:** 1 Faculty of Medicine and Surgery, University of Enna Kore, 94100 Enna, Italy; salvatore.lavalle@unikore.it (S.L.); caterina_gagliano@hotmail.com (C.G.); 2 Clinical and Experimental Radiology Unit, Experimental Imaging Center, IRCCS San Raffaele Scientific Institute, Via Olgettina 60, 20132 Milan, Italy; edo.masiello@gmail.com; 3 Department of ‘Organi di Senso’, University “Sapienza”, Viale dell’Università, 33, 00185 Rome, Italy; giannicola.iannella@uniroma1.it (G.I.); giuseppe.magliulo@uniroma1.it (G.M.); annalisa.pace@uniroma1.it (A.P.); 4 Department of Human Anatomy and Experimental Oncology, Faculty of Medicine, UMONS Research Institute for Health Sciences and Technology, University of Mons, 7022 Mons, Belgium; jerome.lechien@unimons.ac.be; 5 Service of Otolaryngology, Hospital Complex of Santiago de Compostela, 15705 Santiago de Compostela, Spain; christian.calvo.henriquez@gmail.com; 6 Department of Medical and Surgical Sciences and Advanced Technologies “GF Ingrassia”, ENT Section, University of Catania, Via S. Sofia, 78, 95125 Catania, Italy; s.cocuzza@unict.it (S.C.); federicamariaparisi@gmail.com (F.M.P.); 7 Service d’ORL et de Chirurgie Cervico-Faciale, Centre Hospitalo-Universitaire de Montpellier, 80 Avenue Augustin Fliche, 34000 Montpellier, France; 8 Department of Otorhinolaryngology, Alexandria University, Alexandria 21577, Egypt; ahmedyassinbahgat@gmail.com; 9 Department of Head-Neck Surgery, Otolaryngology, Head-Neck and Oral Surgery Unit, Morgagni Pierantoni Hospital, Via Carlo Forlanini, 34, 47121 Forlì, Italy; giovanni.cammaroto@hotmail.com; 10 Department of Anaesthesia and Intensive Care, University Hospital Policlinico-San Marco, 95125 Catania, Italy; luigilavia7@gmail.com; 11 ENT and Audiology Department, University of Ferrara, 44121 Ferrara, Italy; dott.albertocaranti@gmail.com (A.C.); claudio@claudiovicini.com (C.V.)

**Keywords:** obstructive sleep apnea syndrome (OSAS), intermittent hypoxia (IH), oxidative stress, inflammation biomarkers, reactive oxygen species (ROS), nitric oxide (NO), inflammatory cytokines, endothelial dysfunction, antioxidant defense, cellular damage

## Abstract

Background: Obstructive sleep apnea syndrome (OSAS), affecting approximately 1 billion adults globally, is characterized by recurrent airway obstruction during sleep, leading to oxygen desaturation, elevated carbon dioxide levels, and disrupted sleep architecture. OSAS significantly impacts quality of life and is associated with increased morbidity and mortality, particularly in the cardiovascular and cognitive domains. The cyclic pattern of intermittent hypoxia in OSAS triggers oxidative stress, contributing to cellular damage. This review explores the intricate relationship between OSAS and oxidative stress, shedding light on molecular mechanisms and potential therapeutic interventions. Methods: A comprehensive review spanning from 2000 to 2023 was conducted using the PubMed, Cochrane, and EMBASE databases. Inclusion criteria encompassed English articles focusing on adults or animals and reporting values for oxidative stress and inflammation biomarkers. Results: The review delineates the imbalance between pro-inflammatory and anti-inflammatory factors in OSAS, leading to heightened oxidative stress. Reactive oxygen species biomarkers, nitric oxide, inflammatory cytokines, endothelial dysfunction, and antioxidant defense mechanisms are explored in the context of OSAS. OSAS-related complications include cardiovascular disorders, neurological impairments, metabolic dysfunction, and a potential link to cancer. This review emphasizes the potential of antioxidant therapy as a complementary treatment strategy. Conclusions: Understanding the molecular intricacies of oxidative stress in OSAS is crucial for developing targeted therapeutic interventions. The comprehensive analysis of biomarkers provides insights into the complex interplay between OSAS and systemic complications, offering avenues for future research and therapeutic advancements in this multifaceted sleep disorder.

## 1. Introduction

Obstructive sleep apnea syndrome (OSAS) is a widespread and intricate respiratory disorder affecting nearly 1 billion adults aged 30–69 years globally, contingent upon geographical variations [1]. Characterized by recurrent episodes of upper airway obstruction during sleep, OSAS sets off a cascade of physiological events, leading to compromised oxygen saturation, elevated carbon dioxide levels, and recurrent arousals that disrupt sleep architecture [2,3]. This phenomenon results in a range of symptoms, including daytime somnolence, impaired cognitive function, and chronic fatigue, significantly impacting affected individuals’ quality of life. Moreover, OSAS is a recognized contributor to morbidity and mortality, elevating the risk of cardiovascular pathologies [4,5], hypertension, cognitive dysfunction, and an accelerated aging process [6]. The cyclic pattern of intermittent hypoxia in OSAS triggers arterial chemoreceptors, heightening sympathetic nervous system activity [7]. This, in turn, influences vascular reactivity, contributing to the generation of free radicals—highly reactive molecules that interact with nucleic acids, proteins, and lipids, thereby altering cellular metabolism and causing cellular damage. This phenomenon, termed oxidative stress, represents an imbalance between the production of oxygen free radicals and antioxidant capacity, measurable through various biomarkers [8,9]. In addition to oxidative stress, OSAS induces pro-inflammatory factors, leading to the production of cytokines like tumor necrosis factor and interleukins 6 and 8 [9]. These cytokines are implicated in the pathogenesis of atherosclerosis and hypertension, positioning OSAS as an independent risk factor for these conditions [10]. While studies suggest an excess of reactive oxygen species in OSAS, consensus is lacking regarding specific markers to measure and the choice of antioxidants for mitigating the detrimental oxidative effects [11,12]. This review explores the intricate relationship between oxidative stress and OSAS, exploring the impact of intermittent hypoxia on the redox balance and the potential downstream effects on cellular and systemic health. By examining the current literature on oxidative stress in OSAS patients, we seek to shed light on the molecular mechanisms involved and the implications for therapeutic interventions targeting oxidative stress in this sleep disorder. 

## 2. Materials and Methods

### Study Protocol

A comprehensive review of the medical literature from January 2000 to December 2023 was conducted using databases such as PubMed, Cochrane, and EMBASE. We considered several study types, including clinical, preclinical, animal research, ongoing clinical trials, and literature reviews. We considered full-text English articles focusing on the adult population or animal subjects, providing reported values for at least one oxidative stress or inflammation marker. 

The literature search was performed using a combination of key terms specific to the domains of obstructive sleep apnea and oxidative stress. Studies exploring inflammation biomarkers such as protein C reactive, tumor necrosis factor-alpha (TNF-α), interleukin 6 (IL-6), interleukin 8 (IL-8), NADPH oxidase, nitric oxide (NO), asymmetric dimethylarginine (ADMA), arginase, antioxidant system, glutathione, vitamin C, and vitamin E were retrieved. These carefully chosen keywords were pivotal in identifying studies pertinent to the intricate relationship between obstructive sleep apnea and markers indicative of oxidative stress and inflammation. 

## 3. Results

**Figure 1:**
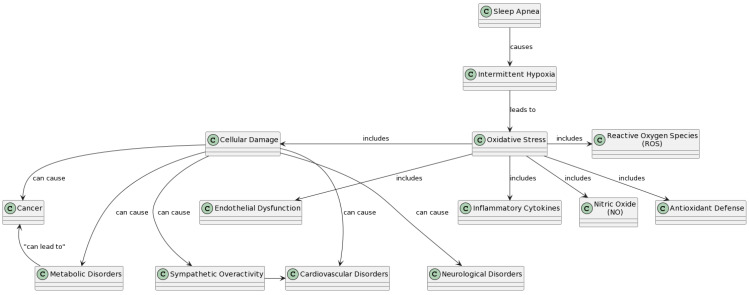
The figure illustrates the intricate network of oxidative stress and inflammation biomarkers in individuals with OSAS. The diagram categorizes the main biomarkers into physiopathogenetic clusters, providing a comprehensive overview of the molecular mechanisms involved in OSAS-related complications. Different clusters represent specific pathways, such as reactive oxygen species biomarkers, nitric oxide regulation, inflammatory cytokines, endothelial dysfunction, antioxidant defense, and cellular damage. The interconnections and associations between these clusters are visually depicted, offering a clear understanding of how OSAS induces oxidative stress, inflammation, and subsequent health complications.

This comprehensive review included 16 research articles in the final analysis. These articles explored several different biomarkers implicated in oxidative stress in patients with obstructive sleep apnea, aimed at proposing new biological markers useful in quantifying systemic inflammation related to OSA. As can be seen in the table below, the included studies were conducted on markers of reactive oxygen species, nitric oxide, inflammatory cytokines, antioxidant defense, and endothelial and organ dysfunction (Table 1). The inherent imbalance between pro-inflammatory and anti-inflammatory factors precipitates heightened oxidative stress, primarily attributed to an upsurge in oxygen free radicals coupled with an inadequate antioxidant capacity [13] (Figure 1). In the complex pathophysiology of obstructive sleep apnea syndrome (OSAS), the intricate interplay of molecular mechanisms begins with the activation of Hypoxia-Inducible Factor 1-alpha (HIF-1α) and nuclear factor kappa-light-chain-enhancer of activated B cells (NF-κB) in response to the chronic intermittent hypoxia that characterizes this condition [14,15,16,17,18]. Such activation is a pivotal adaptive response to hypoxemia, but it becomes maladaptive when repeatedly triggered, leading to a cascade of subsequent events [19,20,21]. The stabilization and activation of HIF-1α upregulate various genes, including those involved in oxidative stress and inflammatory responses, while NF-κB plays a crucial role in the transcriptional activation of pro-inflammatory cytokines [22]. The oscillating oxygen levels drive the generation of reactive oxygen species (ROS), such as superoxide dismutase (SOD), glutathione reduced (GSH), and catalase (CAT), overwhelming the endogenous antioxidant defenses and tipping the balance towards a state of oxidative stress [17]. This oxidative stress, in turn, facilitates the activation of NF-κB, further promoting the release of pro-inflammatory cytokines like tumor necrosis factor (TNF) and interleukins (IL), such as IL-6 and IL-8 [12,18]. These cytokines contribute to systemic inflammation and play a role in the development of endothelial dysfunction, a precursor to atherosclerosis and cardiovascular disease [8,23]. Elevated levels of these markers correlate with the severity of OSAS, typically measured by the apnea–hypopnea index (AHI). Furthermore, the depletion of antioxidant molecules like GSH and the accumulation of oxidized equivalents such as glutathione oxidized (GSSG) reflect the impaired redox state in OSAS patients. The intracellular ratio of GSSG to GSH rises, indicating oxidative stress, while the activity of enzymes like SOD and CAT is often found to be altered, reflecting the body’s attempt to counteract the increased oxidative burden [22]. The results highlight a complex network of interrelated pathways involving HIF-1α and NF-κB activation, ROS production, antioxidant defense compromise, inflammatory cytokine release, and endothelial dysfunction, all contributing to the pathophysiological landscape of OSAS [11,17]. These mechanisms serve as both potential biomarkers for the severity of the disease and targets for therapeutic intervention to alleviate the systemic consequences of OSAS [15]. 

A comprehensive understanding of chronic systemic inflammation involves the quantification of various inflammatory biomarkers present in blood or urine, emanating from nucleic acids, proteins, and lipids. Furthermore, the recurrent cycles of chronic hypoxia/reoxygenation and sleep fragmentation, culminating in an augmented production of reactive oxygen species, circulating cytokines, and adhesion molecules, have been extensively correlated in the literature with cardiovascular, metabolic, and neurodegenerative comorbidities in individuals with OSAS. The interplay of these factors offers insights into the intricate connections between the physiological perturbations associated with OSAS and the development of associated health complications (Table 1). 

### 3.1. Altered Sleep Architecture and Intermittent Hypoxia in Obstructive Sleep Apnea 

Sleep architecture in OSA patients is significantly disrupted due to frequent awakenings or micro-awakenings and chronic intermittent hypoxia (CIH) typical of obstructive sleep apnea [4,34,35,36]. In patients with OSA, CIH and frequent awakenings lead to a significant reduction in the quantity and quality of N3 and REM sleep [37,38,39]. This disruption results in a sleep pattern characterized by excessive transitions between sleep stages, with more time spent in the lighter stages of sleep and less time in the deeper, restorative stages [40,41,42]. Molecularly, the sleep disruptions can impact the expression and regulation of various neurotransmitters and neural pathways that are critical for maintaining sleep stages, particularly the deeper stages of non-REM sleep and REM sleep [43,44]. The lack of restorative sleep further exacerbates the systemic effects of CIH, as it impairs the body’s healing and metabolic processes that are typically more active during these deeper sleep stages. The interplay between CIH-induced molecular pathways and disrupted sleep architecture leads to a cycle of physiological stress and impaired tissue function [45,46,47]. OSA-induced sleep fragmentation sets off a domino effect of sympathetic nervous activation, circadian rhythm disruption, inflammatory pathway engagement, endocrine dysregulation, and oxidative stress, all of which intertwine to contribute to the multi-system impact of this sleep disorder [48,49,50,51,52,53]. The interrelated nature of these mechanisms highlights the importance of addressing sleep quality and architecture in the management and treatment of OSA. Altered sleep architecture in obstructive sleep apnea leads to a complex cascade of pathophysiological events, particularly due to sleep fragmentation, starting with the activation of the sympathetic nervous system [50,51,52]. Each arousal catapults the body into a *‘*fight or flight’ state, increasing heart rate and blood pressure, which, over time, can result in cardiovascular complications and heightened stress responses [53,54,55]. Simultaneously, the disrupted sleep pattern wreaks havoc on the body’s circadian rhythms. These rhythms are essential for regulating not only sleep and wakefulness but also various hormonal outputs such as melatonin and cortisol [5,24,30]. The disarray caused by OSA can lead to mood disturbances and metabolic issues as these hormones become dysregulated. Moreover, as sleep is fragmented, the stages of sleep that usually help to down-regulate pro-inflammatory pathways are interrupted, resulting in elevated levels of inflammatory cytokines like IL-6 and TNF-α [53,54,55,56,57]. This state of chronic inflammation is a contributing factor to systemic health issues and can accelerate atherosclerotic processes [58,59,60]. The repercussions of fragmented sleep and heightened sympathetic activity also extend to endocrine functions. The normal secretion patterns of hormones, including those from the hypothalamic-pituitary-adrenal axis and growth hormone, are altered, contributing to an array of metabolic dysregulations, such as insulin resistance and abnormal appetite control [24]. Furthermore, the oxidative stress burden increases as the body’s antioxidant defenses are compromised due to inadequate restorative sleep, leading to cellular damage and contributing to the risk of developing cardiovascular disease, neurodegeneration, and other oxidative stress-related pathologies [33,43,44]. The association of Hypoxia-Inducible Factor 1-alpha (HIF-1α) with the pathophysiological processes of obstructive sleep apnea (OSA) is a critical element [10]. HIF-1α is instrumental in the body’s adaptive response to the hypoxic conditions characteristic of OSA, which result from repeated airway blockages leading to intermittent hypoxia. Upon a decrease in oxygen levels, HIF-1α stabilizes and accumulates, triggering the transcription of various genes aimed at helping the body adjust to the lack of oxygen [20]. HIF-1α is deeply involved in the molecular pathways that lead to oxidative stress, sympathetic overactivity, and systemic inflammation seen in patients with OSA. It influences the expression of genes associated with angiogenesis, erythropoiesis, and glucose metabolism, and it escalates the production of reactive oxygen species (ROS) by enhancing the expression of enzymes responsible for mitochondrial respiration [22]. This escalation in ROS may exceed the capacity of antioxidant defenses, causing oxidative stress, which subsequently damages DNA, proteins, and lipids within cells [21,26,30]. Additionally, HIF-1α plays a role in the inflammatory response that is characteristic of OSA. It can initiate the transcription of pro-inflammatory cytokines and adhesion molecules, which contribute to systemic inflammation and endothelial dysfunction [40,51]. These mechanisms are pivotal in the onset of cardiovascular diseases, which are frequently seen as comorbid conditions in individuals with OSA. Recognizing the significance of HIF-1α in the pathogenesis of OSA is vital, for it may highlight new therapeutic targets. Modulating HIF-1α signaling pathways could potentially reduce the negative impact of intermittent hypoxia on oxidative stress and inflammation. 

### 3.2. Reactive Oxygen Species Biomarkers

Reactive oxygen species (ROS) biomarkers in OSAS play a crucial role in unraveling the intricate relationship between this sleep disorder and the oxidative stress it induces. The respiratory or oxidative burst orchestrates the generation and release of ROS, including the superoxide anion, hydrogen peroxide, hydroxyl radical, and singlet oxygen [14]. 

Various stimuli, such as the characteristic hypoxia observed in OSAS, can initiate this reaction [15]. Nicotinamide adenine dinucleotide phosphate oxidase (NADPH oxidase), an enzyme, plays a central role in this process, converting free oxygen (O_2_) into superoxide and subsequently triggering the production of other reactive molecules like hydroxide anions, peroxide, hypochlorite, and nitrogen monoxide [16,17]. Importantly, this enzymatic step is responsible for oxidizing biological compounds such as lipids, proteins, and DNA, leading to altered plasma concentrations of associated oxidative markers [18]. Elevated levels of the superoxide anion, a primary ROS, are observed in OSAS. This molecule is a key contributor to oxidative stress, participating in various pathways that can lead to cellular damage [19]. Superoxide anions are also converted to hydrogen peroxide by superoxide dismutase enzymes; while hydrogen peroxide is less reactive and can function as a signaling molecule, in excessive amounts, it contributes to oxidative damage, necessitating the activation of cellular defenses like catalase and glutathione peroxidase for its breakdown. Hydrogen peroxide (H_2_O_2_), another ROS, is produced because of the dismutation of the superoxide anion. Increased H_2_O_2_ levels in OSAS contribute to oxidative stress, affecting cellular components and signaling pathways [20]. The hydroxyl radical (•OH), one of the most potent ROS, is generated through the Fenton reaction. Its presence in OSAS signifies a heightened state of oxidative stress, potentially impacting cellular structures and functions. Singlet oxygen (1O_2_), a highly reactive form of oxygen, can initiate oxidative damage to biomolecules such as lipids, proteins, and DNA [21]. These reactive species are proficient at initiating lipid peroxidation, which compromises cell membranes and creates toxic byproducts that further damage macromolecules. Proteins, vital for myriad cellular functions, are susceptible to structural and functional alterations due to the oxidative modification of amino acids, with consequences that include disrupted enzyme activities and signaling pathways. DNA is not spared; it undergoes oxidative attacks that can result in mutations or even genomic instability, potentially leading to cell death or carcinogenesis. Mitochondria, implicated in both ROS generation and targeting, suffer from oxidative damage that impairs their function, culminating in a dysfunctional energy supply and the release of signals that promote cell death. Specific studies directly addressing singlet oxygen in OSAS are limited but, along with other ROS, may contribute to endothelial dysfunction, inflammation, and tissue damage observed in OSAS patients [22]. ROS-induced endothelial dysfunction can precipitate a series of events that restrict blood flow and further deprive tissues of oxygen, setting the stage for more ROS production. Compounding the problem, ROS can activate matrix metalloproteinases that degrade the extracellular matrix, undermining tissue architecture and stability. The balance between cell survival and death is also tipped, as apoptosis and necrosis pathways are triggered by excessive ROS levels, contributing to organ dysfunction. Even autophagy, a cellular cleanup process, can be thrown into disarray by ROS, leading to the accumulation of cellular debris and dysfunction. Moreover, ROS influence cell signaling pathways, sometimes fostering pathological conditions by promoting aberrant cell proliferation and survival.

### 3.3. Nitric Oxide

Nitric oxide (NO) assumes a multifaceted role in OSAS. Synthesized by nitric oxide synthase (NOS), thanks to the essential amino acid L-arginine. NO regulates vascular tone through vasodilation, but its balance is influenced by OSAS-related factors like intermittent hypoxia. OSAS is associated with endothelial dysfunction, and the reduced bioavailability of NO, often linked to oxidative stress and inflammation, may contribute to cardiovascular complications, the mediating vasodilating effect, and platelet aggregation [4,33]. The interaction between NO and ROS in the context of intermittent hypoxia can lead to the formation of peroxynitrite by interaction with superoxide or the action of dimethylarginine (ADMA), potentially contributing to oxidative stress. At elevated levels, it disrupts the synthesis of NO by diminishing the activity of the enzyme dimethylarginine dimethylaminohydrolase, resulting in increased levels of ADMA [12,45,46,47,48]. In recent times, several investigations have delved into the levels of NOx in individuals with OSAHS. Nevertheless, the findings are inconclusive. Kapusuz et al. [24] observed notably elevated plasma NO levels in OSAS patients in contrast to the control group, whereas Canino et al. [25] did not observe any distinction in NO levels between OSAS subjects and their healthy counterparts. A recent meta-analysis conducted by Wu et al. found that OSAS was significantly related to serum or plasma NO levels and that serum or plasma NO levels in OSAS patients are lower than in controls [30]. Studies suggest a connection between OSAS, diminished NO bioavailability, and increased cardiovascular risks, with impaired NO-mediated vasodilation contributing to hypertension and atherosclerosis [4,32,33]. NO may also play a role in sleep regulation, and changes in its levels could contribute to the sleep disturbances observed in OSAS [38]. Despite its involvement in various physiological processes, the precise mechanisms and therapeutic implications of NO in OSAS remain areas of active research.

### 3.4. Antioxidant Defense

The antioxidant defense system includes various components such as enzymes (e.g., superoxide dismutase, catalase, and peroxidase) and non-enzymatic molecules (e.g., glutathione, vitamin C, and vitamin E) [13,22]. These antioxidants work synergistically to neutralize ROS and prevent oxidative damage to cellular components like lipids, proteins, and DNA. Individuals with OSAS exhibit an imbalance between the production of oxidative agents and the compensatory action performed by the antioxidant system, known as total antioxidant capacity (TAC) [7,18,39,40]. Superoxide, a vital cellular oxidizing agent, undergoes dismutation catalyzed by the superoxide dismutase (SOD) enzyme family, leading to the dissociation of the superoxide anion into molecular oxygen and hydrogen peroxide in healthy individuals [41,42,43,44,45]. However, OSAS patients have been reported to have lower plasma levels of SOD [30,31]. Catalase (CAT) plays a crucial role in mitigating oxidative stress by facilitating the breakdown of hydrogen peroxide into water and molecular oxygen [39]. The primary function of glutathione peroxidase (GPx) is to catalyze the reduction of hydrogen peroxide (H2O_2_) and organic hydroperoxides, utilizing reduced glutathione (GSH) as a substrate [45]. This enzymatic reaction helps prevent the accumulation of harmful ROS within cells and tissues [40]. The glutathione system, which includes GPx, acts as a first line of defense against oxidative damage by neutralizing peroxides and maintaining the cellular redox balance [49]. A study conducted by Asker et al. demonstrates that OSAS patients had lower levels of CAT and GPX [46]. Interestingly, a correlation was detected between CAT and GPX levels and polysomnographic indices. Both correlated directly with the AHI, but glutathione peroxidase levels were inversely correlated with the mean duration of apnea. Literature suggests that molecules like glutathione, vitamin C, and vitamin E contribute to ameliorating oxidative stress in OSAS patients, especially in conjunction with continuous positive airway pressure (CPAP) therapy [49,55]. Additionally, oxidative stress is linked to sleep disturbances in OSAS patients, and the intake of antioxidants has been shown to enhance sleep quality [7]. Sales et al. discovered reduced antioxidants in OSAS patients, indicating a correlation between antioxidants and neuropsychological alterations in obstructive sleep apnea [33]. Specifically, they observed decreased levels of vitamin E (*p* < 0.006), superoxide dismutase (*p* < 0.001), and vitamin B11 (*p* < 0.001), along with increased homocysteine levels (*p* < 0.02).

### 3.5. Inflammatory Cytokines

In individuals affected by OSAS, encompassing both children and adults, the presence of chronic intermittent hypoxia induces a systemic inflammatory response. Additionally, key inflammatory cytokines such as interleukin-8, tumor necrosis factor-alpha, and interleukin-6 exhibit upregulation, potentially linked to the activation of the nuclear factor pathway [46]. OSAS is characterized as a low-grade chronic inflammatory respiratory condition, as the repetitive episodes of chronic intermittent hypoxia during sleep instigate an anti-inflammatory cascade. These cytokines, often elevated in the serum of OSAS patients, can exert widespread effects on multiple organ systems. The upregulation of these cytokines in OSAS is thought to be closely associated with the activation of the nuclear factor kappa-light-chain-enhancer of activated B cells (NF-κB) pathway. NF-κB is a transcription factor that plays a critical role in the inflammatory response. Under normal conditions, NF-κB is sequestered in the cytoplasm by the inhibitor IκB. However, during episodes of hypoxia, IκB is phosphorylated and degraded, freeing NF-κB to translocate into the nucleus, where it can initiate the transcription of various inflammatory genes, including those for IL-8, TNF-α, and IL-6. These inflammatory mediators contribute to the pathophysiology of OSAS by promoting leukocyte recruitment, inducing the expression of adhesion molecules on endothelial cells, and elevating the production of reactive oxygen species (ROS). Moreover, pro-inflammatory cytokines may modulate metabolic processes, influence the hepatic production of acute-phase reactants, and contribute to the development of insulin resistance.

#### 3.5.1. Tumor Necrosis Factor-α

Tumor necrosis factor-alpha (TNF-α) serves as a pivotal proinflammatory cytokine in the intricate immunological landscape of OSAS. OSAS, characterized by recurrent episodes of hypoxia and reoxygenation, manifests an augmented inflammatory milieu marked by heightened systemic levels of TNF-α. Evidence suggests that TNF-α levels positively correlate with the severity of OSAS [47]. The nuanced interplay of TNF-α with other inflammatory mediators in OSAS contributes to the complexity of the inflammatory cascade, with cumulative effects implicated in the development of cardiovascular comorbidities commonly observed in individuals with OSAS. TNF-α plays a significant role in promoting atherosclerosis, inducing the expression of cellular adhesion molecules, and facilitating the adhesion of leukocytes to the vascular endothelium [48,49]. Elevated circulating levels of TNF-α have been correlated with early atherosclerotic signs in healthy middle-aged individuals [46,50,51,52,53]. Furthermore, these levels serve as predictive markers for coronary heart disease and congestive cardiac failure. In the context of OSAS, TNF-α concentration is elevated compared to healthy subjects, underscoring its potential contribution to cardiovascular risks in OSAS patients. Remarkably, continuous positive airway pressure (CPAP) treatment demonstrates the capacity to normalize TNF values in OSAS individuals, suggesting a potential avenue for mitigating the inflammatory impact associated with this sleep disorder [29,30,31]. 

#### 3.5.2. Interleukin-8 

Interleukin-8 (IL-8), acknowledged as one of the most potent inflammatory cell chemokines, plays a crucial role in initiating systemic inflammation in OSAS and associated cardiovascular conditions. IL-8 functions by inducing myeloperoxidase release from neutrophils and recruiting inflammatory cells, contributing to a sustained inflammatory response. Akyol et al. reported that IL-8 binding to specific receptors on neutrophil surfaces leads to cell deformation, degranulation, and increased production of reactive oxygen species [53]. This process may activate arachidonic acid through lysosomal secretion, resulting in heightened vascular permeability, plasma protein exudation, and subsequent tissue damage, atherosclerosis, and other diseases [54]. Recent meta-analysis findings emphasize that individuals, both children and adults, with OSAS exhibit significantly elevated IL-8 concentrations, with IL-8 levels positively correlating with the severity of OSAS indicated by the apnea–hypopnea index (AHI) and being linked to obesity and ethnicity [55].

#### 3.5.3. Interleukin-6

Interleukin-6 (IL-6) is a multifunctional cytokine with several biological activities, such as the proliferation of T lymphocytes, the differentiation of B lymphocytes, and the stimulation of immunoglobulin secretion [51,58]. Moreover, IL-6 plays a role in regulating the natural sleep patterns associated with the circadian secretion pattern. In individuals with OSAS, particularly during episodes of intermittent hypoxia and reoxygenation, there is an upregulation of IL-6 as part of the systemic inflammatory response [59,60,61,62]. A meta-analysis performed by Nadeem et al. indicates elevated levels of interleukin-6 (IL-6) in patients with OSAS compared to control individuals [46]. Furthermore, a recent metanalysis performed by Imani et al. confirms the significant correlation between IL-6 and the AHI, indicating a potential link between IL-6 and the severity of OSAS, and has also highlighted a positive correlation between IL-6 production and body mass index [59]. 

### 3.6. Endothelial Dysfunction

A potential early sign of vascular disease is endothelial dysfunction [60]. Research indicates that individuals with OSAS who have not experienced vascular issues previously exhibit endothelial dysfunction [61]. Unfortunately, the specific mechanisms triggering the development of endothelial dysfunction in OSA remain unclear [62]. Exposure to harmful cellular risks, such as oxidative stress, may result in endothelial dysfunction [63,64,65], leading to a reduction in its ability to dilate blood vessels, an elevation in proinflammatory and prothrombotic reactions, and abnormal regulation of vascular growth [66]. OSAS induces intermittent hypoxia, triggering oxidative stress and inflammation. This, in turn, prompts the release of proinflammatory cytokines, such as IL-6 and TNF-α, and elevates C-reactive protein levels (CRP) [46,55]. These inflammatory mediators lead to an endothelium with proinflammatory tendencies and subsequent endothelial dysfunction. This dysfunctional state is characterized by an elevation in the expression of cell adhesion molecules (CAMs), including E-selectin, vascular cell adhesion molecule-1 (VCAM-1), and intercellular adhesion molecule-1 (ICAM-1) [67,68]. These molecules facilitate the adhesion and migration of leukocytes into the vessel wall, a crucial step in the initiation of atherosclerosis [69,70]. A dysfunctional endothelium also exhibits diminished nitric oxide levels, a reduction that may result from an elevated CRP level, leading to the downregulation of endothelial nitric oxide synthase (eNOS) expression and bioactivity [71]. Furthermore, the reduction in NO levels may be attributed to the presence of superoxide anions arising from an imbalance between ROS synthesis and antioxidant systems, leading to oxidative stress [72]. These pathophysiological mechanisms associated with OSAS potentially contribute to the development of cardiovascular events, such as systemic hypertension and other cardiovascular diseases, and are a key factor in developing atherosclerotic plaques [73].

### 3.7. Cellular Damage and Organ Dysfunction

In individuals with OSAS, repetitive airway collapse and obstruction due to various pathological factors result in recurrent apnea, periodic arousal during sleep, intermittent hypoxia (IH), and sleep fragmentation. These core processes trigger various cellular [71] and molecular mechanisms, including increased sympathetic nerve activity [69], metabolic dysregulation [58], systemic inflammation [73], oxidative stress, and endothelial dysfunction [60]. These mechanisms, identified as pathogenic in clinical and experimental models, contribute to OSAS-related complications across different systems [61,62,63,64]. 

#### 3.7.1. Cardiovascular Disorders

Notably, OSAS is strongly associated with cardiovascular complications, including systemic hypertension, arrhythmias, coronary artery disease, and stroke [50,57,66,73]. The link between OSAS and hypertension is particularly significant, with up to 80% of patients with resistant hypertension potentially suffering from OSAS [73]. The increase in sympathetic nerve activity, driven by ROS, is a prominent feature of OSAS and is implicated in OSAS-related cardiovascular issues [74,75]. Oxidative stress, inflammation, and molecular mechanisms play crucial roles in developing cardiocerebrovascular diseases in OSAS patients [76]. In addition, intermittent hypoxia (IH) and recurrent arousals, likely through mechanisms involving oxidative stress and activation of Hypoxia-Inducible Factor 1 (HIF-1), result in sympathetic overactivity in patients with obstructive sleep apnea (OSA). The effects of this overactivity include elevated catecholamine levels, systemic hypertension, changes in ventricular repolarization, and cardiac remodeling. These physiological changes contribute to the cardiovascular burden often observed in individuals with OSA [60,61,62,63,64]. The complex interplay of these factors underscores the importance of early intervention and treatment of breathing disorders during sleep to prevent cardiovascular morbidity [77]. In addition, the recurrent changes in intrathoracic pressure associated with obstructive sleep apnea (OSA) provoke an augmented venous return to the heart, which in turn can cause an overload of the right ventricle. Moreover, the intrathoracic pressure dips below that of the external structures surrounding the heart, which increases the afterload on the left ventricle. This heightened afterload can impair the heart’s systolic and diastolic functions. Over time, these pressures may lead to a chronic dilation of the left atrium, which could have significant implications for cardiac health and function. Animal studies have provided significant insights into the cardiovascular consequences of obstructive sleep apnea (OSA) and the molecular mechanisms underlying these effects. In various animal models, OSA is simulated through induced intermittent hypoxia, mirroring the oxygen desaturation-reoxygenation cycles seen in human OSA. These studies have shown that such hypoxic episodes can lead to sympathetic nervous system activation, oxidative stress, and systemic inflammation—all factors contributing to cardiovascular pathology. Key molecular pathways include activation of the sympathetic nervous system and downstream signaling processes such as those mediated by HIF-1α, which have been implicated in the development of hypertension and atherosclerosis. In rodent models, intermittent hypoxia has been shown to lead to endothelial dysfunction, vascular remodeling, and a propensity for arrhythmogenesis, providing a mechanistic basis for the association between OSA and increased cardiovascular risk. In humans, the relationship between OSA and cardiovascular events has been extensively studied through randomized clinical trials and observational cohorts. The evidence suggests that OSA is independently associated with an increased risk of hypertension, coronary artery disease, heart failure, and arrhythmias, most notably atrial fibrillation. continuous positive airway pressure (CPAP) therapy has been the cornerstone of OSA management, with several trials demonstrating its efficacy in reducing apneic events and improving the quality of sleep. However, its role in the secondary prevention of cardiovascular events remains controversial. While some studies have shown that CPAP treatment can lower blood pressure and reduce the risk of recurrent cardiovascular events, others have not found a significant benefit in terms of cardiovascular outcomes. This has led to an ongoing debate in the field, with some experts suggesting that the heterogeneity in patient populations, varying adherence to CPAP treatment, and differences in baseline cardiovascular risk may contribute to these conflicting results. Future studies with rigorous design, perhaps focusing on personalized medicine approaches to identify those most likely to benefit from CPAP, are needed to clarify its role in cardiovascular risk reduction among patients with OSA.

#### 3.7.2. Neurological Disorders

Prolonged exposure to IH in patients with OSAS has profound effects on various central nervous system (CNS) functions, resulting in severe neurocognitive and behavioral deficits. OSAS is associated with a decline in cognitive functions, including memory, executive function, and comprehension, as well as mood disturbances, insomnia, and excessive daytime sleepiness [77,78]. Animal studies indicate that IH induces neuronal injury, inflammation, and astrocyte activation in the rat brain, leading to impaired cognitive performance in tasks such as the Morris water maze test [78,79]. Clinical studies in OSAS patients reveal cognitive impairments in attention, delayed memory function, and executive function, which are correlated with the severity of OSAS. Structural and functional alterations in brain anatomy, including decreased gray matter in various regions, provide indirect evidence of brain damage in OSAS patients [76,80]. The brain, being sensitive to hypoxia, experiences oxidative stress, inflammation, and neuronal damage due to IH. The involvement of ROS, oxidative stress, inflammatory damage, and microglial activation contributes to neuronal apoptosis and/or necrosis, leading to OSAS-related cognitive impairments. The NF-κB, TNF-α, CRP, IL-1β, IL-6, and cyclooxygenase-2 (COX-2) pathways are implicated in neuroinflammation and cognitive dysfunction in OSAS [78,79]. Microglia, as major inflammatory cells in the CNS, mediate oxidative stress and inflammation, and their activation is associated with neurotoxicity [80]. The NF-κB-mediated JNK and p38 MAPK pathways play crucial roles in hippocampal injury and cognitive dysfunction [17]. Additionally, brain-derived neurotrophic factor (BDNF) and excitotoxic neurotransmitters such as glutamate contribute to OSAS-related CNS damage [81]. The accumulating evidence highlights the intricate relationship between inflammation and cognitive impairment in OSAS, suggesting potential links with neurological disorders that warrant further investigation.

#### 3.7.3. OSAS, Obesity, and Metabolic Disorders

Emerging evidence from animal models of OSAS suggests that IH is independently linked to metabolic dysfunction. OSAS demonstrates an independent association with insulin resistance, implying its potential role in the development of type 2 diabetes and metabolic syndrome, characterized by obesity, insulin resistance, hypertension, and dyslipidemia [82]. Clinical studies have revealed significantly higher levels of fasting blood glucose and insulin resistance in OSAS patients, with the severity of OSAS correlating with increased insulin resistance [83]. This association extends to non-obese patients, and AHI has been identified as an independent risk factor for insulin resistance and type 2 diabetes [84]. IH-induced oxidative stress and inflammation in OSAS contribute to insulin resistance, with inflammatory factors inhibiting insulin receptors and the phosphorylation of insulin receptor substrates. IH also impacts glucose metabolism by reducing glucose uptake in muscles, affecting pancreatic β-cell function, and increasing sympathetic tone, thereby disrupting glycemic and insulin homeostasis. OSAS is further implicated in lipid abnormalities, elevating total cholesterol, triglycerides, LDL, and VLDL levels [85]. 

Obstructive sleep apnea (OSA) is closely intertwined with obesity, a condition that itself is a well-established pro-inflammatory state. The high prevalence of overweight and obese individuals among OSA patients complicates the understanding of the systemic inflammation observed in OSA. Adipose tissue in obese individuals is not merely a storage depot for excess calories but an active endocrine organ that secretes a variety of cytokines and inflammatory mediators, such as TNF-α, IL-6, and C-reactive protein (CRP). These mediators contribute to chronic, low-grade systemic inflammation. In OSA, intermittent hypoxia and sleep fragmentation further exacerbate this inflammatory milieu. However, distinguishing the inflammation due to OSA from that due to obesity can be challenging, as both conditions independently contribute to systemic inflammation and share common pathophysiological pathways. As such, the inflammation observed in OSA patients may be compounded by the presence of obesity, making it a confounding factor in the assessment and management of inflammation in OSA. This overlap implies that the therapeutic strategies targeting OSA should also consider the management of obesity to effectively mitigate the compounded inflammatory state. Treatment with CPAP may positively influence lipid profiles. IH-associated changes in leptin and adiponectin levels contribute to insulin sensitivity and metabolic homeostasis [86,87,88]. Although the exact relationship between OSAS and metabolic diseases is still debated, recognizing their strong association is crucial for early detection and intervention. Further research is needed to elucidate specific mechanisms and address controversies in this complex relationship.

#### 3.7.4. OSAS, Oxidative Stress, and Cancer

In recent years, accumulating circumstantial, epidemiological, clinical, and experimental evidence has strongly suggested a notable impact of OSAS on tumorigenesis and tumor development. A comprehensive multicenter cohort study involving cancer-free OSAS patients revealed a significant association between nocturnal hypoxemia and overall cancer incidence [89]. Moreover, individuals under 45 years old with severe OSAS demonstrated a markedly increased risk of various cancer types compared to the general population [90]. Epidemiological investigations further confirmed a link between OSAS and elevated cancer-related mortality, revealing a dose–response relationship between OSAS severity and cancer-specific mortality. This association spans over a 22-year follow-up period, where severe OSAS was associated with nearly a fivefold risk of death from cancer [91,92,93,94]. OSAS is implicated in raising the incidence of specific tumor types, including lung cancer, breast cancer, prostate cancer, nasopharyngeal tumors, and melanoma. Notably, in certain tumors, exposure to IH, mimicking the oxygenation pattern induced by OSAS during sleep, has been shown to promote the growth, invasion, and metastasis of lung cancer, colon cancer, and melanoma [92].

### 3.8. OSA Treatment Effectiveness on Inflammation and Oxidative Stress

CPAP remains the gold standard treatment for OSA. Numerous studies have demonstrated the effectiveness of CPAP in reducing systemic inflammation, a key player in the pathogenesis of atherosclerosis and cardiovascular disease [95,96,97,98]. Inflammatory biomarkers, such as C-reactive protein (CRP), TNF-α, and interleukins (IL-6 and IL-8), have been shown to decrease significantly with compliant use of CPAP therapy [99]. Oxidative stress, which contributes to endothelial dysfunction and subsequent cardiovascular disease, is also mitigated by CPAP [100]. Markers of oxidative stress, such as malondialdehyde (MDA) and nitric oxide (NO) levels, exhibit notable improvements with CPAP use [101]. The impact of CPAP on reducing inflammation and oxidative stress has profound implications for comorbid conditions. For instance, cardiovascular risk factors such as hypertension and arrhythmias are markedly improved with effective CPAP therapy, likely due to the reduction in sympathetic nervous system activity and improved vascular endothelial function [102]. Similarly, CPAP use has been linked to improvements in insulin sensitivity and lipid profiles, reducing the risk for metabolic syndrome and type 2 diabetes [103,104]. The reduction of inflammation and oxidative stress through the treatment of OSA has a favorable impact on several comorbid conditions [105]. For instance, the cardiovascular benefits of reducing these pathological processes are substantial, leading to a decrease in the incidence of myocardial infarction, stroke, and heart failure [106]. Additionally, improvements in metabolic outcomes, such as better glucose control and lipid metabolism, can significantly reduce the risk of diabetes and contribute to weight loss [107]. Cognitive benefits are also noteworthy, as untreated OSA is associated with an increased risk of cognitive decline and dementia. By reducing inflammation and oxidative stress, which are implicated in neurodegeneration, treatments for OSA may also preserve cognitive function and reduce the risk of neurocognitive disorders [108]. Other respiratory indices have been assessed to identify predictors of OSA treatment. The study by Fernandes et al. analyzed the relationship between mean oxygen saturation (SpO_2_) and inflammatory markers in OSA patients treated with CPAP [109]. They found that subjects with a lower mean SpO_2_ (<95%) had a higher inflammatory profile, including a higher number of leukocytes, a higher number of neutrophils, a higher number of basophils, and an elevated concentration of C-reactive protein. These results suggest that SpO_2_ levels may play a role in predicting the inflammatory status and treatment outcome of OSAS subjects. Conversely, the study conducted by Ming et al. reported that TNF-α levels were negatively correlated with the mean and lowest oxygen saturation levels (MSaO_2_ and LSaO_2_). Additionally, they observed a positive correlation between IL-8 levels and AHI, as well as morning systolic and diastolic blood pressure, while IL-8 levels were negatively correlated with MSaO_2_ and LSaO_2_. These results suggest that TNF-α and IL-8 may be variably involved in the inflammatory and cardiovascular consequences of obstructive sleep apnea [110]. In the study conducted by Tauman et al., they found that children with moderate-severe sleep-disordered breathing (SDB) had increased plasma levels of IL-6 compared to controls, and this increase was statistically significant (*p* = 0.03). In particular, the levels of IL-6 were positively correlated with the apnea-hypopnea index (AHI) (r = 0.28, *p* = 0.003) and negatively correlated with the lowest oxygen saturation levels (SpO_2_ nadir) (r = −0.24, *p* = 0.02). Additionally, the study revealed that children with SDB exhibited severity-dependent increases in plasma C-reactive protein (CRP) and IL-6 levels, regardless of their obesity status. Although less frequently, outcomes on oxidative stress, inflammation, and different specific biomarkers have been evaluated in other types of treatment. MADs are oral appliances designed to advance the mandible and, consequently, the base of the tongue, enlarging the airway space to reduce apneic events [111]. While CPAP is more effective in reducing the apnea–hypopnea index (AHI), MADs offer a viable alternative for patients with mild to moderate OSA or those who are non-compliant with CPAP. Studies have shown that MADs can lead to improvements in inflammatory markers similar to those seen with CPAP, albeit to a lesser extent in some cases [112]. The reduction in oxidative stress with MAD use, while still a topic of ongoing research, has promising preliminary results, suggesting that they can offer cardiovascular protective effects [113,114]. Surgical options for OSA aim to address anatomical abnormalities contributing to airway obstruction. These procedures range from uvulopalatopharyngoplasty (UPPP) to more complex surgeries such as maxillomandibular advancement (MMA) [34,35]. The impact of surgery on inflammatory and oxidative stress markers is less clear than with CPAP or MADs, primarily due to the variability in surgical techniques and individual patient anatomy [115]. However, successful surgical outcomes that lead to a significant reduction in AHI do correlate with a decrease in systemic inflammation and oxidative stress [116]. Despite the potential benefits, surgical treatments are often considered a last resort due to their invasive nature and associated risks. When surgery successfully reduces or eliminates apneic events, it can have a significant impact on reducing the overall inflammatory burden and the risk of cardiovascular disease [117]. Moreover, surgery may provide a permanent solution for selected patients, which can be particularly appealing compared to the need for ongoing treatment with CPAP or MADs.

### 3.9. Future Perspectives for Sleep Apnea Biomarkers

The burgeoning field of biomarker research in OSA is uncovering novel pathways and targets that could revolutionize the diagnosis and treatment of this complex disorder. Fan et al. have shown that NAD+ biosynthesis reduction may lead to mitochondrial dysfunction and vascular endothelial injury, which are critical in the pathogenesis of OSA [63]. Under chronic intermittent hypoxia (CIH), the study found a decrease in NAD+ biosynthesis due to inhibited NAMPT enzyme activity, which led to mitochondrial dysfunction in endothelial cells, characterized by reduced ATP and mitochondrial membrane potential, impaired respiratory chain activity, increased oxidative stress, and compromised vascular function. Supplementing with nicotinamide mononucleotide (NMN) reversed the mitochondrial and endothelial dysfunction caused by CIH. However, endothelial damage induced by oxidized low-density lipoprotein (ox-LDL) did not show involvement of the NAD+ pathway and was not mitigated by NMN supplementation. Similarly, Chen et al. have identified that the long, non-coding RNA FKSG29 plays a pivotal role in regulating oxidative stress and endothelial dysfunction in OSA, suggesting a new molecular target for intervention [64]. The authors reported that FKSG29 and certain pro-oxidant genes were upregulated in OSA patients, while anti-oxidant genes were downregulated compared to primary snorers. In vitro, knocking down FKSG29 in cells exposed to intermittent hypoxia with re-oxygenation (IHR) reduced reactive oxygen species production, apoptosis, and abnormal gene expression associated with oxidative stress, and these protective effects were negated by concurrently knocking down miR-23a-3p. The research suggested that targeting the FKSG29/miR-23a-3p/IL6R pathway could be a novel therapeutic strategy for OSA-induced endothelial dysfunction. Promising data are also present on the role of miRNA as a novel biomarker for OSA patients. The work of Fadaei et al. supports the potential utility of circulating miRNAs, specifically miR125a, miR126, and miR146a-5p, as biomarkers for endothelial dysfunction in OSA patients, which could serve as non-invasive diagnostic tools [65]. The transition from physiological adaptation to pathological maladaptation in response to chronic intermittent hypoxia, a hallmark of OSA, is discussed by Arnaud et al., providing insights into the systemic effects of the disorder [66]. Nguyen et al. highlighted the role of peripheral inflammation due to sleep fragmentation, suggesting that inflammation biomarkers could be a key to understanding and treating OSA-related comorbidities [93]. In particular, the authors found that acute sleep fragmentation in male C57BL/6J mice induced a swift activation of the hypothalamic-pituitary-adrenal axis, increasing serum corticosterone levels within 1 h and persisting up to 24 h. Instead, a peripheral inflammatory response was evidenced by elevated pro-inflammatory gene expression in the heart from 1 h of ASF and a delayed increase in serum IL-6 concentration after 6 h. Collectively, a future can be envisioned where a comprehensive biomarker panel derived from genetic, molecular, and inflammatory markers could be developed, facilitating a more nuanced approach to OSA management, enabling earlier diagnosis, better risk stratification, and more precise targeting of therapies to ameliorate the multifactorial consequences of the disorder.

## 4. Study Limitations

Notwithstanding the fact that this study offers a thorough synthesis of the most recent data about biomarkers in OSAS, some limitations must be noted. One major drawback is that many of these biomarkers are not measured using uniform methods and assays across studies, which could explain part of the variation in reported findings. Few studies used longitudinal follow-up, and the majority had sample sizes that were quite small. The cohorts’ limited applicability to women and other age groups resulted from their predominance of middle-aged male participants. Confounding variables were frequently not sufficiently taken into consideration, including underlying comorbidities, drugs, and lifestyle choices. Synthesizing biomarker data is further complicated by the variety of OSAS populations, which vary in severity, symptoms, and co-occurring medical illnesses. To validate findings, more high-quality research with reliable techniques, larger sample numbers, confounder adjustment, and longitudinal evaluations are warranted. Assay and biomarker panel standardization would enable cross-study comparability. It is necessary to include a variety of patient demographics that reflect the heterogeneity of OSAS in the real world. Although this analysis offers a solid foundation, its shortcomings point to the need for additional thorough investigation to improve our knowledge of the mechanisms of inflammation and oxidative stress that underlie the pathophysiology and clinical consequences of OSAS. Filling up these gaps would increase the possibility of biomarkers being translated into better diagnosis, prognosis, and treatment for OSAS.

## 5. Conclusions

Obstructive sleep apnea syndrome (OSAS) significantly impacts health, driving a range of complications through oxidative stress and inflammation. This disorder affects multiple physiological processes, contributing to cardiovascular, neurological, and metabolic disorders, and may increase cancer risk. The pathophysiology of OSA involves a complex interaction of factors, including sympathetic activation, endothelial dysfunction, hypoxia-induced metabolic imbalance, and increased inflammatory and proatherogenic activity. The intermittent hypoxia characteristic of OSAS leads to oxidative stress, systemic inflammation, and subsequent multi-organ dysfunction. Cardiovascular issues, cognitive decline, and metabolic syndrome are closely linked to OSAS, necessitating multidisciplinary research and clinical approaches. The potential role of OSAS in cancer progression also highlights the need for further sleep-related oncology research. Despite progress, gaps remain, particularly in identifying biomarkers and effective antioxidant therapies. Understanding OSAS’s molecular mechanisms is critical for developing targeted treatments and integrating sleep medicine into comprehensive patient care.

## Figures and Tables

**Table 1:** Properties of oxidative stress indicators in individuals with obstructive sleep apnea syndrome (OSAS). Abbreviation: SOD, superoxide dismutase; GSH, glutathione reduced; GSSG, glutathione oxidized; AHI, apnea–hypopnea index; TNF, tumor necrosis factor; NADPH, nicotinamide adenine dinucleotide phosphate; IL, interleukin; CAT, catalase.

Authors	Study Characteristics	Outcome
Reactive Oxygen Species		
Liu H.G., Zhou Y.N., Liu K. et al. (2010) [17]	30 OSAS patients vs. 23 healthy controls.	The mRNA levels of NADPH oxidase p22phox in sputum samples significantly increased in individuals with OSAS (*p* < 0.05).
R. Schulz, S. Mahmoudi, K. Hattarm et al. (2000) [21]	18 OSAS patients vs. two control groups of 10 healthy volunteers and 10 patients without OSAS.	The release of superoxide demonstrated a marked increase in each comparison (*p* < 0.01).
	Nitric Oxide	
Duchna H.W., Guilleminault C., Stoohs R.A. et al. [23]	23 male OSAS patients and 12 male healthy controls.	Patients with OSAS exhibit impaired endothelium-dependent NO-mediated vasodilation (*p* < 0.001).
Kapusuz Gencer Z., Özkiriş M., Göçmen Y. et al. [24]	36 OSAS patients vs. 22 healthy controls.	There is a positive correlation between plasma NO levels and AHI.
Canino B., Hopps E., Calandrino V. et al. [25]	48 OSAS patients vs. 31 healthy controls.	Across the entire OSAS subject group, no significant difference in NO was identified when compared to the control group.
Wu, Z.H., Tang, Y., Niu, X. et al. [26]	Metanalysis of a total of 7 eligible studies, including 250 OSAS patients and 158 non-OSAS patients).	OSAS exhibited a significant association with serum or plasma NO levels (WMD = −11.66, 95% CI −17.21 to −6.11; *p* < 0.01), indicating that serum or plasma NO levels in OSAS patients are lower than those in controls.
	Inflammatory Cytokines	
Lin C.C., Liaw S.F., Chiu C.H. et al. (2016) [27]	35 patients with moderately severe to severe OSAS vs. 20 healthy controls	TNF-α levels were elevated (*p* < 0.01).
Li X., Hu R., Ren X., He J. (2021) [28]	Metanalysis of a total of 25 eligible studies, including 2301 participants and 1123 controls to evaluate the association between serum IL-8 concentration and OSAS.	Correlation between serum IL-8 concentration and OSAS, revealed that both adults and children with OSAS exhibited higher serum IL-8 concentrations compared to controls (SMD = 0.997, 95% CI = 0.437–1.517, *p* < 0.001; SMD = 0.431, 95% CI = 0.104–0.759, *p* = 0.01).
Ifergane G., Ovanyan A., Toledano R. et al. (2016) [29]	The final analysis incorporated 43 individuals experiencing acute stroke and sleep apnea.	There was a positive correlation between AHI and IL-6 (ρ = 0.37, *p* = 0.02).
Wu M.F., Chen Y.H., Chen H.C. et al. (2020) [30]	The final analysis incorporated 100 participants, comprising 63 individuals with normal to moderate OSAS and 37 with severe OSAS.	There was a significant interaction effect on IL-6 levels for all OSAS severity and sex (*p* = 0.030). Additionally, IL-6 levels were higher in the obese group than in the non-obese group, irrespective of OSAS severity and sex (*p* = 0.000).
Yokoe T., Minoguchi K., Matsuo H. et al. (2003) [14]	A total of 30 individuals diagnosed with OSAS and 14 obese participants serving as control subjects.	IL-6 levels were significantly higher in patients with OSAS compared to the control group (*p* < 0.05).
	Antioxidant Defense	
Tian Z., Sun H., Kang J. et al. (2022) [31]	Metanalysis of a total of 14 eligible studies, including 1240 patients and 457 controls.	The circulating SOD levels in patients with OSAS were significantly lower than those in the control group (SMD = −1.645, 95% CI = −2.279 to −1.011, *p* < 0.001).
Ntalapascha M., Makris D., Kyparos A. et al. (2012) [32]	18 patients with severe OSAS and 13 controls were included in the study.	The overnight ratio of GSH/GSSG and the levels of GSH were significantly different from controls (*p* = 0.03 and *p* = 0.048, respectively).
Sales L.V., Bruin V.M., D’Almeida V. et al. (2013) [33]	14 patients with obstructive sleep apnea vs. 13 controls.	Vitamin E exhibited lower levels (*p* < 0.006), SOD showed a decrease (*p* < 0.001), vitamin B11 demonstrated a decline (*p* < 0.001), and homocysteine concentrations increased (*p* < 0.02). Serum concentrations of vitamin C, CAT, GSH, and vitamin B12 remained unaltered.
Simiakakis M., Kapsimalis F., Chaligiannis E. et al. (2012) [18]	66 total subjects were referred (42 patients with OSAS vs. 24 controls).	The antioxidant capacity levels in OSAS patients were significantly lower (*p* = 0.004).
Mancuso M., Bonanni E., Lo Gerfo A. et al. (2012) [15]	41 untreated patients with a new diagnosis of OSAS vs. 32 healthy subjects.	The total GSH levels were significantly lower in OSAS patients than controls (95% CI for the mean 0.389–0.449 nmol/μL vs. 0.574–0.713 nmol/μL; *p* < 0.0001).

## Data Availability

No new data were created or analyzed in this study. Data sharing is not applicable to this article.
